# Supplementary material for: The interactome of intact mitochondria by cross-linking mass spectrometry provides evidence for coexisting respiratory supercomplexes
Source: Mol Cell Proteomics. 2017 Dec 8;17(2):216–32. doi: 10.1074/mcp.RA117.000470 (PMC5795388; doi:10.1074/mcp.RA117.000470)
Supplement: Supplemental Data [file supp_RA117.000470_133922_0_supp_23978_fzffwf.zip › spectra_annotation/mito_DR_spectra_annotation/416-1-19-1-15-1.pdf]

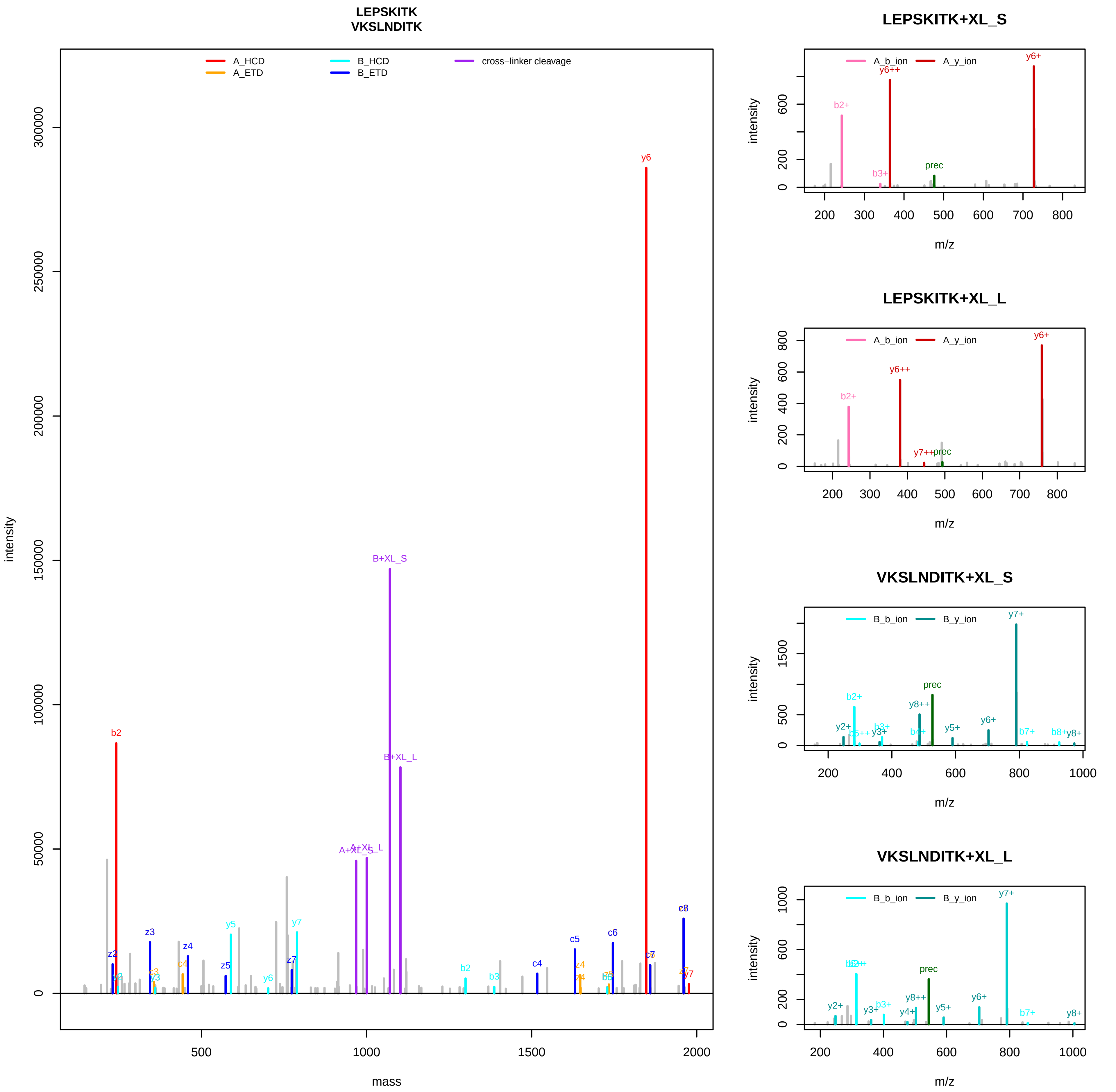

LEPSKITK
VKSLNDITK
A_HCD
A_ETD
B_HCD
B_ETD
cross−linker cleavage
intensity
mass
y6
b2
B+XL_S
B+XL_L
A+XL_S
A+XL_L
c8
y5
y7
z3
z4
z2
z5
z7
c4
c5
c6
c7
b2
b3
y6
LEPSKITK+XL_S
LEPSKITK+XL_L
VKSLNDITK+XL_S
VKSLNDITK+XL_L
A_b_ion
A_y_ion
B_b_ion
B_y_ion
y6+
y6++
b2+
b3+
prec
y7++
y7+
y8++
y5+
b7+
b8+
y8+
m/z
